# A conceptual model of functional health literacy to improve chronic airway disease outcomes

**DOI:** 10.1186/s12889-021-10313-x

**Published:** 2021-01-30

**Authors:** Iraj Poureslami, Noah Tregobov, Jessica Shum, Austin McMillan, Alizeh Akhtar, Saron Kassay, Kassandra Starnes, Maryam Mahjoob, J. Mark FitzGerald

**Affiliations:** 1 University of British Columbia and VGH Divisions of Respiratory Medicine, Centre for Lung Health, Vancouver Coastal Health Research Institute, Gordon and Leslie Diamond Health Care Centre, 2775 Laurel Street, Vancouver, BC V5Z 1M9 Canada; 2 Queen’s University, 94 University Avenue, Kingston, ON K7L 3N6 Canada; 3 Respiratory Medicine Division, Department of Medicine, University of British Columbia, Gordon and Leslie Diamond Health Care Centre, 2775 Laurel Street, Vancouver, BC V5Z 1M9 Canada; 4 School of Medicine, Brookfield Health Sciences Complex, University College Cork, College Road, T12 AK54, Cork, IE-M Ireland; 5 Faculty of Health Sciences, Simon Fraser University, 8888 University Drive, Burnaby, BC V5A 1S6 Canada; 6 Faculty of Law, University of Windsor, 401 Sunset Ave, Windsor, ON N9B 3P4 Canada

**Keywords:** Health literacy, COPD, Asthma, Chronic airway disease management, Measurement tool, Functional skills

## Abstract

**Background:**

Current conceptual models of health literacy (HL) illustrate the link between HL and health outcomes. However, these models fail to recognize and integrate certain elements of disease management, health system factors, and socio-demographic factors into their framework. This article outlines the development of Chronic Airway Disease (CAD) Management and Health Literacy (CADMaHL) conceptual model that integrates the aforementioned elements and factors into a single framework.

**Methods:**

Information obtained during the following stages informed the development of our model: (1) a systematic review of existing CAD HL measurement tools that apply core HL domains; (2) patient-oriented focus group sessions to understand HL barriers to CAD self-management practices; (3) key-informant interviews to obtain potential strategies to mitigate CAD management barriers, and validate disease self-management topics; (4) elicited the perspectives of Canadian respirologist’s on the ideal functional HL skills for asthma and COPD patients.

**Results:**

Throughout the study process many stakeholders (i.e., patients, key-informants, and an international HL advisory panel) contributed to and reviewed the model. The process enabled us to organize the CADMaHL model into 6 primary modules, including: ***INPUT,*** consisting of four HL core components (access, understand, communicate, evaluate,) and numeracy skills; ***OUTPUT*,** including application of the obtained information; ***OUTCOME*,** covering patient empowerment in performing self-management practices by applying HL skills; ***ASSESSMENT,*** consisting of information about functionality and relevancy of CADMaHL; ***IMPACT,*** including mediators between HL and health outcomes; ***CROSSCUTTING FACTORS,*** consisting of diverse socio-demographics and health-system factors with applicability across the HL domains.

**Conclusions:**

We developed the CADMaHL model, with input from key-stakeholders, which addresses a knowledge gap by integrating various disease management, health-system and socio-demographic factors absent from previous published frameworks. We anticipate that our model will serve as the backbone for the development of a comprehensive HL measurement tool, which may be utilized for future HL interventions for CAD patients.

**Trial registration:**

NCT01474928- Date of registration: 11/26/2017.

**Supplementary Information:**

The online version contains supplementary material available at 10.1186/s12889-021-10313-x.

## Background

The Canadian Expert Panel on Health Literacy [1] and Calgary Charter on Health Literacy [2] define health literacy (HL) as a person’s ability to *access, understand, communicate, evaluate and apply health information to make informed decisions for their health* [1, 3]. Historically, researchers have also considered numeracy to be a HL skill; however, since numeracy is a variable applicable to all core HL domains, it is typically assessed across the domains rather than independently [4–6].

Several reports indicate that the prevalence of low HL is a significant and growing public health concern [7–10]. Such an issue has the potential to widen existing health inequities in acquiring care services and health information, especially among disadvantaged populations, including older adults [11–13], minority groups [1, 3], individuals of low socio-economic status, and people suffering from chronic diseases (including those with CAD) [14–16]. Individuals with low HL face barriers to adequately accessing health services and may encounter challenges when communicating with health care providers or making informed health decisions, both of which are crucial elements of disease self-management practices [17–19]. Inadequate HL is also associated with increased rates of unnecessary hospitalization and emergency department visits [20, 21], poor medication adherence [22, 23], lower quality of life [24, 25], and increased mortality [26, 27].

Despite various studies assessing the link between HL and CAD outcomes [28–35], the bulk of these studies are descriptive or cross-sectional in design, and they solely establish associations between HL and health status. As a result, they fail to establish the long-term impact of HL on health outcomes, and are also limited by the use of inadequate measurement tools [13, 36–38]. From a methodological point of view, HL measurement tools reported in the literature have received criticism for their inability to incorporate the full spectrum of key factors influencing an individual’s HL skills [39, 40]. The models used to inform the development of existing HL tools for CAD management fail to encompass all 5 essential HL domains and numeracy comprehensively [37]. In addition, the models do not consider the impact of internal (e.g., such as person’s home culture, beliefs, attitude, worldview, cognition, and psychological issues) and external factors (e.g., socio-environment and health system issues) on the attainment of HL skills, including added behavioural components and accessibility of health information and care services [3, 4, 41]. The deficiencies in current HL conceptual models provide limited understanding of essential factors influencing a patient’s self-management practices [42, 43]. Recent debates have been called for developing a comprehensive HL model for chronic disease management, that not only enables researchers and clinicians to adequately assess HL skills, but also informs the need for practical interventions, aiming to empower patients to better self-manage their chronic condition(s) [44–47]. The call was acknowledged by many researchers globally that recommended more work is needed to: 1) clarify how HL is conceptualized at different levels of practice [44]; 2) further demonstrate the causal link between HL and disease self-management outcomes [45, 46]; and 3) integrate personal attributes and social support into HL models to facilitate patient engagement in the disease management process [47, 48].

As HL is a rapidly evolving and expanding concept [49, 50], there has been a call-to-action to develop appropriate frameworks to comprehensively measure its core components. Several models have been reported in the literature describing HL as a multidimensional construct that improves an individual’s skills related to accessing, understanding and using health information to make informed decisions about one’s health [51–53]. For instance, McCormack et al. [54, 55] developed a model that presents HL as an individual-level attribute that is affected by predisposing factors or socio-environmental aspects of the target population (e.g., culture and beliefs). Other models focus merely on mediator elements (factors that influence a relationship) between HL and health outcomes, and how the model can be used as a screening tool [43, 51]. Therefore, there have been significant challenges in applying the existing models in clinical practice, as their approaches are primarily theoretical (research-based) and lack clinical significance and applicability [56].

The conceptualization of HL should consider and integrate key constructs and measures across the core domains to improve data capture, facilitate intervention development, and enable benchmarking [43]. An Institute of Medicine report concluded that there is a need to increase understanding of factors affecting patient’s HL skills, and how these skills may influence their self-efficacy to engage in disease self-management practices [7, 47]. Studies have also suggested that HL may be conceptualized as an empowering tool to increase patient engagement in disease management, and an effectual and influential preventive measure [57–59]. Therefore, the conceptualization of information that is derived from the insights of key-informants and knowledge-users and the creation and operationalization of corresponding items for each domain are necessary to develop an accurate and valid HL model [60]. To the best of our knowledge, no study has been reported in the literature that conceptualizes HL as a preventive measure and empowerment tool that can further enable an individual to engage in risk-perception and behavioural modification practices. There is also a lack of reported involvement of community members during the design and evaluation stages of such models. Additionally, there has been a noted failure to consider the full spectrum of intrinsic factors (e.g., beliefs, worldviews, perceptions, and practices) that may influence decision-making, navigation of health system complexities, and the attainment of the requisite skills condition self-management skills.

**Fig. 1:**
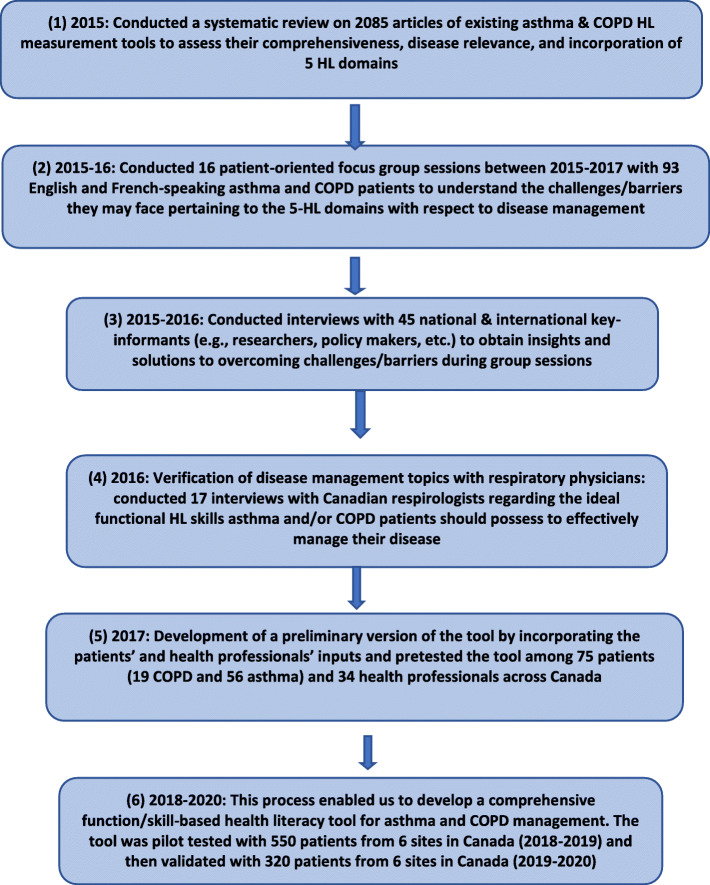
Conceptualization asthma and COPD disease management health literacy tool: Needs assessment, pretesting, pilot and validation phases

**Fig. 2:**
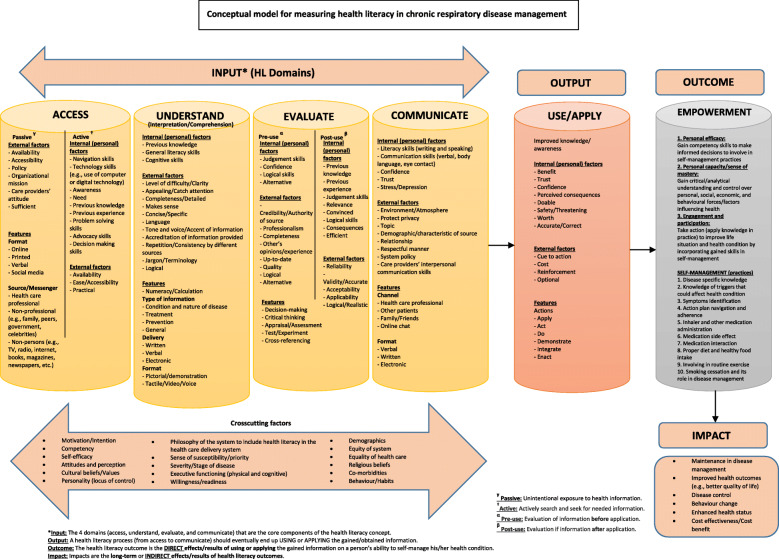
Conceptual model for measuring health literacy (HL) in Chronic Respiratory Disease (CRD) management

In this article, we summarize the conceptualization process (Fig. 1. Multistage study process) of our Chronic Airway Disease Management and Health Literacy (CADMaHL) model (Fig. 2. Conceptual model for measuring health literacy (HL) in CAD management). The model incorporates insights from patient participants, health care professionals, and HL researchers regarding personal attributes, external barriers and facilitators to self-management, and an individual’s capabilities to apply HL skills in the decision-making process. We summarize methods used to identify key constructs in the conceptual framework and to analyze data elicited from stakeholders’ perspectives. In the Results section, we further elaborate on the conceptualization process and present the developed CADMaHL model. We then discuss the potential implications and include information on how the CADMaHL model can be applied to guide future research, evaluation, and interventions on CAD management.

## Methods

Institutional ethics approvals were obtained from each of the collaborating centres across Canada. The study protocol was registered at ClinicalTrials.gov (NCT01474707). All participating patients provided written informed consent, and key-informants and respirologists provided either electronic or written informed consent prior to participating in the different stages of this study.

### Identifying key constructs

A multi-design study of multiple stages was applied to conceptualize and develop our HL model through the following needs assessment stages (Fig. 1). (1) A systematic review of 2800 articles was conducted in 2015 to assess the comprehensiveness and disease- relevance of factors included within existing HL tools, and whether they incorporated the five core HL domains in their structure [37]. (2) 16 patient-oriented focus groups were conducted across Canada with 93 adult male and female asthma and COPD patients from 2015 to 2016 to understand the challenges/barriers pertaining to the five HL domains with respect to self-management practices, as well as to identify the most important disease management topics that patients were interested to receive information on [61]. (3) 45 key-informant interviews conducted in-person/ telephone/Skype with health care professionals, researchers, and policymakers from Canada, the US, the UK, and Australia in 2016 to obtain their insights on possible solutions to overcome the challenges expressed by focus group patients as well as to help validate the disease self-management topics identified during focus group sessions [62]. (4) 17 interviews with Canadian respirologists were conducted in 2016 regarding the ideal functional HL skills asthma and/or COPD patients should possess to effectively self-manage their disease [63, 64]. All of the aforementioned focus group and interview content and questions were developed solely for use in this study. An additional pdf file contains the questions asked during patient focus groups, and key-informant and respirologist interviews (see Additional file 1).

### Theoretical development

The conceptualization of our model included information derived from the insights of key-informants from our global and national knowledge hubs, and patients. Eliciting and integrating the perspectives of these individuals was critical in the operationalization of corresponding items for each domain, and, therefore, necessary to develop a framework for our model. A key-informant (health literacy expert) from our knowledge hub indicated: *A framework is an essential component in the development of a function-based assessment tool to measure patients’ HL, as it provides structure for choosing the most important skills and content to be assessed. Involvement of the patients, community and professionals in the conceptualization of the tool will help to construct a credible, reliable framework to ensure using right tool to collect proper information to produce outcome that have both content and face validity with reliable results.* To address the current knowledge gaps in existing models, we relied upon the following purposefully selected models and theories, as the foundational platforms for knowledge synthesis and model construction. A Validity-Driven approach [65] was used in the development of the CADMaHL conceptual model. Firstly, the Chronic Care Model (CCM) [66] was used for integration of key-informants and knowledge users on the research team (patients, health care professionals, and health care decision-makers) to incorporate their guidance on topics and items selection. Secondly, the Interactive Systems Framework (ISF) [67] served as the foundation for integrating of patients’ and key-informants’ understanding of potential factors that influence HL into our model– specifically related to disease self-management practices. Thirdly, the COM-B theory (capability, opportunity and motivation) [68] was applied to describe possible causal mechanisms related to the application of gained information and attained HL skills to action and behavior change required to implement self-management practices. The incorporation of the CCM, ISF, and COM-B theory collectively facilitated targeted identification and integration of internal and external factors in our model to address barriers and identify skills required for proper disease management among asthma and COPD patients.

### Data analysis

Audio recordings and notes from the focus group sessions, discussions, and interviews were transcribed verbatim. For those sessions taking place in French language, professional translators translated the transcriptions into English. Two team members (J.S. & I.P.) and one research assistant with experience in qualitative research analysis used NVivo software (QSR International, version 12) to code the data and conduct thematic analysis. Details of the content analysis method applied in this study have previously been reported [61–64]. Qualitative information was extracted, coded, and sorted into categories/sub-categories with similar statements to develop the framework [69, 70]. The main objective was to obtain a large verbatim sample to conceptualize HL. Content analysis helped to identify viewpoints about the role of beliefs and perceptions as well as the system-related factors relevant to accessing and using information and services for their disease self-management practices. Following an inductive approach to data analysis, the large sample of verbatim quotes and observations from stakeholders across the four stages yielded six primary modules: (a) **INPUT**: four core HL domains (access, understand, evaluate, and communicate) and numeracy skills. (b) **OUTPUT**: the use domain, pertaining to application of the obtained information in making informed decisions for self-management. (c) **OUTCOME**: patient empowerment and confidence in performing self-management practices. (d) **ASSESSMENT**: HL assessment tools and tests to monitor outcomes and facilitate benchmarking. (e) **IMPACT**: mediators between HL and health outcomes resulting in behavioural change, better disease control, and improved health status. (f) **CROSSCUTTING FACTORS**: diverse factors with applicability across the HL domains (e.g., cultural beliefs/values, personality, self-efficacy, etc.).

## Results

### Conceptualization process

Different researchers have indicated that a corresponding HL model should focus on functional HL skills and capabilities [44, 48]. Throughout the four aforementioned stages of our study, it was highly recommended by both patients and professional groups that the HL model should comprises both internal and external factors that affect HL skills as well as health-related actions. In addition, there was a consensus to emphasise that it is of primary importance to consider the ways the healthcare system is responsible for creating an environment that enables people to freely access needed information and services. A key-informant indicated: “*You should develop a framework for health literacy that could follow a life course determinant model and focus on functionality...Also important are constructs from behavioural science, such as the self-efficacy and health belief model.*” Another key-informant emphasised “*A conceptual framework for health literacy should cover three major features of our society’s reality: a) cultural practices and views; b) systemic issues with health care delivery system, and c) equitable access to services and resources … .. It seems to me all these essential elements contribute to health outcomes and well-being of diverse community members, including patients with chronic disease.*” From our systematic review, we learned that existing CAD HL measurement tools, and the frameworks used to inform their development, fail to properly account for an individual’s ability to use HL skills in real world health contexts [43]. The studies applying these academic skills have operationalized HL as literacy skills in a medical setting and measured those skills through standardized reading tests [11, 28, 30, 32, 58]. As a result, interventions based on existing HL frameworks have primarily aimed to make information easier to understand, by reducing the cognitive demand, rather than emphasizing empowerment and engagement of patients. Reading and understanding information are important parts of functional HL, but they offer an incomplete picture of a person’s capacity to actively navigate, find and use health information and services correctly or engage in self-management practices. In contrast, functional HL becomes a concept that describes the practical application of a wide range of cognitive and non-cognitive skills in real-world contexts- such as problem-solving, communication, interpersonal skills, and lifelong learning skills. As a result, we conceptualized the CADMaHL model for asthma and COPD patients that can help researchers to include elements of functional HL across the 5 core HL domains and numeracy with applicability transcending the clinical setting and incorporating a variety of influential internal and external factors [7, 71, 72]. See Table 1 for more quotes from study participants.

**Table 1:** Participant, HL researcher, key-informant, & health care professional quotes

Conceptualization Process	
“*You need to focus on functional health literacy, which is a concept that describes the practical application of a wide range of cognitive and non-cognitive skills in real life, rather than a single literacy skill in a clinical setting.*” (Key-Informant)	
“*Frame your model to develop a functional health literacy tool, which is the outcome of intervention rather than the independent variable and captures how people use literacy for their health. Your framework for health literacy should follow a life course determinant model.”* (HL Researcher)	
“*Most of the HL work myopically focuses on the patient side. It’s time to focus on health care professionals and the system’s health literacy. Doing so, we need to work with public health professionals, work with journalists. Need to measure both sides of the partnership and the context [patients and care provider]. Analyze the tasks, tools and systems.*” (HL Researcher)	
Proposed CADMaHL Model	
“… *well, your framework should cover two different things: I think if you want to measure ability to obtain and understanding the concepts, information, and services, I would go with the first four domains, accessing, understanding, communicating and evaluating. If you want to measure people’s agency and confidence in using information to make change, I’d go with the Use domain at the end of the spectrum.*” (HL Researcher)	
“*Your definition [Calgary Charter definition of HL] seems adequate except from the addition of a link between health literacy competence and changes in lifestyle or disease management practice.*” (HL Researcher)	
“*To me, health literacy means ‘enacting’ or ‘putting into practice’ the knowledge for living healthy. I would like to see a tool asking patients [to] illustrate how they would actually enact or use information they obtain into self-management practice – this is health literacy!”* (Respiratory Educator)	
Module 1.1 Access	
*“Whether individuals are competent to access needed services, handle transitions, and find relevant information, which indeed are the navigation skills”.* (Key-Informant)	
*“Maybe you should add navigation skills into the accessing information domain to see if you can assess your patients’ ability to seek and find needed information.”* (Key-Informant)	
*“Lots of people think they can go and find information [themselves]; everything they need to know about medications now, yet 80% of the stuff you find on the web is crap. So, unless they go and get good advice from their doctor, they are going to be misled by internet and exposed to unnecessary risk.”* (Patient)	
Module 1.2 Understand	
*“I use plain language and then the teach-back method to get the patients to show me what they retain. For example, when I am teaching an inhaler, I have placebos for them to use to show me what I have just taught them. So, sometimes they get to understand that way”.* (Respiratory Educator)	
*“[In using prednisone] some things might not be clear to people because of jargon [used by the doctor] which might make this more difficult”.* (Patient)	
*“Still, I do not know the different types of inhalers; I know one helps me faster than the other, but I don’t really understand the difference between the puffers. Information in English that are verbally translated to a foreign language are often difficult for patients (non-English speaking) to understand”.* (Patient)	
Module 1.3 Evaluation	
*“If after following the doctor’s information and instruction I am actually able to manage my chronic condition, and actually able to see that the amount of flare ups have been decreasing, I would apply it in my daily routine when I get positive reinforcement.”* (Patient)	
*“How do I know whether the information is useful? I’ll try it out, what, like, if the doctor has said and if it works then I’ll follow. So, it is a trusting of my doctor’s knowledge and also my feeling that it works for me.”* (Patient)	
*“… it [information] has to be able to allow you to use it to make some sort of a strategy and then be able to evaluate if it’s working, if it’s not working, if it needs to be adjusted before you apply it again.”* (Patient)	
*“The person’s ability to find relevant health information and support is the first step to self-manage their disease. It highly depends on patient’s needs to assess the information they receive (with a recognition that relevance of the information depends on their current personal needs and changing contexts of their lives) and use this understanding in decision-making which will lead to actions which are health enhancing.”* (HL Researcher)	
“*In order for information to be useful, first of all, you have to identify with it. It has to be pertinent to you. It has to be accurate. It’s something that I’ll look at and it might raise a question or two that I can take to my doctor.*” (Patient)	
Module 1.4 Communication	
*“My doctor should convince me why I have to take a new medication, but there are other people around me that always ask ‘why [are] you taking this medication?’ ‘Don’t listen to them [doctors].’ ‘That’s not good for you, but you have to decide.’ That’s the kind of communication challenge that I’m normally juggling, what should I do? Should I listen to my very good friend [*sic*] -- my family next to me for many years? Should I listen to my doctor because I trust my doctor?”* (Patient)	
*“I feel that gender differences are a barrier for communication with health care providers.) I know Muslim women have to go to a woman doctor because they are not allowed to have another man see any of them. It affects some cultures because they can’t you know. It’s just the way it is”.* (Patient)	
*“People don’t like telling their doctors [disease-related] things. I feel the honesty is not there. I am not excluded from that.”* (Patient)	
Module 2. Output	
“*I just kept on smoking into the 1990s and then I quit. That’s when it was explained to me clearly [by my doctor] that I wouldn’t live too long with COPD if I didn’t stop smoking.*” (Patient)	
“*A health-literate person is capable to enact or put into practice the information (actually practice knowledge) for living a healthy lifestyle. Therefore, I think change or reinforcing healthy lifestyle practices should be the main output of health literacy.*” (HL Researcher)	
“*I followed instructions [given by my doctor] and monitored my asthma mainly because of my experience in using it and it worked; so, it was the feeling of need and trust to apply it.*” (Patient)	
“*Well, I believe to integrate information into lifestyle, one needs readiness for change and motivation to use the information.*” (Patient)	
“*Application of health information in routine disease management practice is a self-reflexive action component whereby the person possessing the health information uses it by taking action on their own behalf for the purpose of changing and improving their health. The patient should have enough skills to use health information more directly to make judgments about what to do or not to do.*” (Respiratory Researcher)	
“*I think the reason patients use the information and incorporate it in their disease management is because of its safety; that’s the information and services is being safe to apply; like if they see positive results from something I’ve told them, whatever it may be like, you know, if you take these inhalers properly [for] 6 to 8 weeks, your shortness of breath will improve, and if they see those results then they’re more likely to be willing to be receptive to other information [received from me] and actually use it. There is a clear recognition that if they use information, their lives are going to be better.*” (General Practitioner)	
Module 3. Outcome	
“*If you don’t evaluate a health outcome relative to HL, you’re selling yourself short. Better access and comprehension should lead to better outcomes. HL is not meant to be normative, and doesn’t predict compliance, but should, overall lead to an improvement in the aggregate.*” (HL Researcher)	
“*Health literacy is one of the most powerful tools we have to empower people.*” (Respiratory Doctor with HL Knowledge)	
“*Empowerment is a key element of health literacy. It includes not only health promoting behaviour but also the ability to perform primary and self-care and, also, motivate patients to ask questions.*” (Respiratory Doctor with HL Knowledge)	
“*I think HL is empowerment of obtaining and understanding health information and utilization of the information to make sound decisions (health-enhancing decisions). Patients need reliable and user-friendly information about how to stay in good health and the effects of lifestyle on their health.*” (Respiratory Doctor)	
“*One of the most obvious impacts of improved HL is an empowered individual with basic skills to self-assessment, self-management as well as awareness of the changes happening in their health. Altogether may reveal the level of effective application (functionality) of gathered information in their real-life situations revealing their skills to act to improve health.*” (Health Literacy Researcher)	
“*HL should empower people with self-care skills, assertiveness skills and problem-solving skills*.” (Respiratory Doctor)	
Module 5. Impact	
“*We need a broad and integrative approach which will be messy and sometimes changes people’s lives, as I believe health literacy is not merely increased knowledge but it should be eventually ended with a change in behaviour.*” (Respiratory Researcher)	
*“[In measuring the impact of health literacy] Multiple sectors need to be engaged and messages must be crafted and supported using data and language that resonates with each target audience”.* (Health care Policy Maker)	
*“It is important that health literacy supporters be prepared for capitalizing ‘windows of opportunity’ by demonstrating the powerful contribution health literacy can make to health promotion, disease prevention and care”.* (Health Literacy Researcher)	
Module 6. Crosscutting Factors	
“*… [Patients] are less inclined to ask questions due to their cultural beliefs and that makes it harder for them to follow instructions or feel confident with asking questions as well.”* (Clinician)	
“*Some individuals are intuitive in terms of how they apply their experience in understanding the information and some others are more analytical and they both have different temporal demands on how they absorb the information and make sense of it in their routine life.*” (Policymaker)	
“*If they don’t see the priority of using information for their health, its use is pathetically low and they are not going to engage in it. That’s because most people’s lives are so crap and using the information that looks boloney to them isn’t going to help them with anything. So, we can sit in our offices and put all these messages out, but unless the person’s context allows them to use it, that is they are safe to use it or they need to use it, then they see there is actually a need to act on it.*” (Respiratory Doctor)	

### The proposed CADMaHL model

The CADMaHL model is a multidimensional framework for HL and related intervention areas to improve CAD outcomes. The model encompasses six modules (as mentioned above) which describe the process of obtaining HL skills, the appropriate application of skills in the decision-making process, and the impact of improved HL on disease management and overall health (Fig. 2). In our model, HL domains are divided into two components: a) INPUT, consisting of the navigation and procurement of information: access, comprehension, evaluation, and communication domains; and b) OUTPUT, consisting of the application/use of obtained information in the decision-making process, as outlined below.

The six modules comprising the CADMaHL model are explained below:

- **3-2a. Input:** Includes four HL domains and numeracy. These domains encompass how an individual actively navigates and obtains health information, understands and evaluates this information, and communicates with others about their health issues.
- 3-2a.i *Access*. This was one of the most debated domains among both the patient and professional groups because it was often stated that health information should initially be provided by the health care system in a simple, effective way, which is both accessible and available. Therefore, it is important to consider the bidirectionality of the access domain (i.e., resource provision by providers and access by patients). It was suggested that we consider the challenges presented to patients in accessing information that is relevant, accessible, available and acceptable in our model (i.e., ability to access available quality information/resources). A key-informant stated, “*I learned by experience that the information needs to be accessible to patients. Even if it’s accessible to them but they don’t understand the language, it does not make sense, so it should be user friendly and should be accessible and available.*” Our previous studies also indicate that an individual’s need for health information is highly dependent on what health-related demands they face (e.g., contracting a particular disease) and whether they are exposed to information unintentionally, or intentionally while navigating and searching for information themselves [60–64, 73]. Therefore, we considered access-related skills in our model to be a two-sided balance: (1) *passive access* to information (e.g., unintentionally received from their physician during an appointment without asking for it, learned in conversation from friends or family members) and (2) *active access*, which is their ability to know where to look and ability to proactively seek and find the information that they need (e.g., visiting health-related websites, asking the physician to provide specific information). A patient mentioned the times needed for active and passive access, “*The provider’s information at the very first visit has to be very clear about when someone should rely on what they can find themselves and when they should consult the professional who can help them with the task.”* Quotes on the ‘Access’ domain are summarized in Table 1-1.1.
- 3-2a.ii *Understand.* Most patients expressed challenges with understanding information (provided in oral or written format) related to the use of medical jargon or complex terminology during interactions with care providers or other sources, particularly regarding symptom recognition and the treatment process. One patient indicated, “*My doctor has a tendency to use big words, I’m not that smart, and sometimes I don’t hear things properly so he repeats it for me and writes it down for me, but I [need] him to explain it to me in a way that I understand.*” Another patient identified that a barrier to fully understanding and comprehend was a limites time during the visit between the patient and care provider: *“My doctor [had to] explain my disease and action plan to me very quickly. I guess she thought I was understanding her, but until I went back home to read the action plan and I realized I didn’t understand exactly what does it mean (sic)...I had to go back and find out information because she did that rather quickly.”* Physicians also discussed the same challenges expressed by the patients: “*I think we as clinicians have to be careful to use laymen’s terms and not so much the medical terminology.*” Among other skills, patients expressed numeracy skills (the ability to calculate numerical information) as necessary for an individual to understand and apply information provided in the health care system. For quotes on the ‘Understand’ domain, please refer to Table 1-1.2.
- 3-2a.iii *Evaluation.* The capacity to make inferences based on available information and the ability to select reliable health information sources and comprehend the relevance of the information to their own health issues were components suggested by key-informants for inclusion within the evaluation module. In addition, to evaluate the applicability of the obtained information or instruction, participants identified two key components of the evaluation and validation process: 1) evaluation of the obtained information before using it (*pre-application*) and 2) after using it (*post-application*). During pre-application evaluation, there was consensus among patients and professionals that perceived credibility and trustworthiness of the information source, which may be influenced by a patient’s biases towards different information sources, were the main factors that prompted individuals to act on or apply the information/instruction. A common sentiment among HL researchers and clinician scientists is that HL is not simply about the medical knowledge that a patient can acquire. Rather, it is the sum of all sources of information that the patient comes across and evaluates; most of the time the patient does not solely rely on the information shared by health professionals, they may seek resources from sources of varying reliability (e.g., the internet, friends/family). Therefore, key-informants suggested including the accuracy, consistency, relevancy, and source (i.e., credibility) of information into our model. A patient stated, “*Well, for me it’s going back to getting the information from a source, usually a trusting source, then go and research it from 20 different reputable sites, and then it’s worked the majority (sic) of it for me because once I’ve researched it enough and I feel comfortable and part of it is your intuition too that comes into play”.* For the post-application evaluation, many patients indicated they would continually apply the information in their routine disease self-management process if they had positive experiences after their initial use of the information. The ability of patients to discern quality information from poor information across a wide variety of sources/inputs is imperative to their disease self-management and health outcomes. More quotes on the ‘Evaluation’ domain can be found in Table 1-1.3.
- 3-2a.iv *Communication.* Many patients and professionals indicated that HL is influenced by interactions with care provider(s) or others who may have shared experiences or some knowledge about the disease. A HL researcher mentioned, “*Respectful communications between provider and patient leads to successful interactions. The mismatch is what is driving the poor outcomes.*” The participants also identified different barriers to proper communication between the patient and care provider. For instance, a respiratory educator emphasized the importance of using proper communication channels to provide critical information to patients, “*First, present important information such as risk information in ways that are accessible to people who communicate with different language than English or French. Second, we need effective vehicles for communication particularly risk information to patients...making sure that it’s lay language that is used.*” The information should also be presented in a manner that is culturally and linguistically appropriate for the patient. Additional quotes on the ‘Communication’ domain can be found in Table 1-1.4.
- **3-2b. Output:** This module focused on a patient’s ability to act on the obtained information and services to perform self-management practices (e.g., using learned disease management strategies to prevent an exacerbation). Although different measures of HL refers to skills related to the understanding and communication of health information [3, 4, 12–14], there must be a purpose for obtaining the health information [73]. This was evident in the feedback from the patients and professionals in our study, who suggested that health information should be used to make sound health decisions and practice health-promoting behaviours. Therefore, to feel fully empowered and health literate, a patient must have the ability to put knowledge into practice [42]. Participants introduced a clear definition of ‘using’ information: *adapting and applying information from trusting source(s) into daily life for disease management*. Patients also indicated that when they received relevant and easy to understand instructions or information about their chronic disease from trusted sources (e.g. their doctor); they were more likely to apply it to their disease self-management. A patient mentioned, “*I got the actual action plan and my doctor explained it in a way that I understood, and I would use it … well, I learned this will help me to prevent more severe flare ups that is why I will use it.*” However, application of the obtained information into an individual’s daily routine, outside of the clinical setting, was not always straightforward. Motivation to navigate information and apply the information was expressed by both patient and professional groups as a necessary aspect of disease management. Another patient mentioned, “*I’ve read all the stuff I received from hospital people. I have listened to doctors. I understand everything about it. Nevertheless, when it comes to actually doing it regularly and keeping where you should be, I have faltered many times and not sticking with it. So, I think I need something to convince me to take it and apply it in my disease control process.*” Patients must first understand the reasons for applying certain health information into their daily lives before they are prompted to do so, and care providers can facilitate this process. Quotes on the ‘Output’ module can be found in Table 1-2.
- **3-2c. Outcome:** This module explains how HL contributes to patient empowerment (providing patients with the right knowledge and confidence to take care of their disease management) and self-efficacy (a patient’s belief that they can control their own disease management process) to influence successful achievement of health care goals. Self-management practices for CAD patients included: disease specific knowledge, knowledge of triggers that could affect health condition, symptom identification, action plan navigation and adherence, inhaler and other medication administration, medication side effects, medication interactions, proper diet and healthy food intake, involvement in routine exercise, and smoking cessation and its role in disease self-management. Many key-informants believed that HL should empower patients to take control as the main caretaker in their disease management. A HL researcher indicated: *“The empowerment skill, as an outcome of HL, I think, will help patients to be proactive and self-confident...I think HL is empowerment of understanding health information and utilization of the information to make sound decisions.”* Patients must feel that they are in the driver’s seat of the disease management process, and be capable to act as the driver in this process. Quotes on the ‘Outcome’ module can be found in Table 1-3.
- **3-2d. Assessment:** The input of patients and health professionals at different stages of the study enabled us to determine measurable aspects of HL that may inform interventions and HL measurement tools. Throughout the process, we learned from patients and key-informants that HL assessments using a self-evaluated approach (where patients report their perceived ability to act in hypothetical health-related situations) may not provide an accurate representation of an individual’s skills due to reporting and self-desirability bias. Key-informants recommended testing the functional HL abilities of patients by assessing their true ability to act in situations, using real-world passage-based scenarios. A HL researcher suggested, *“In your model, you need to assess patient’s functional skills, navigation capability, understanding instruction/ information, and motivation to apply the knowledge into practice with [a] measurement tool.”*
- **3-2e. Impact:** This module defines the mediators between HL and health outcomes, resulting in behavioural change, disease control, and improved health status and outcomes. It demonstrates the process of improving disease self-management outcomes as the results of improved HL skills. One HL researcher expressed, “*Learning more about the expectations and demands on a person with chronic disease will help to conceptualize the model to assess the change in person’s behaviour and lifestyle.*” Empowering patients through targeted interventions aiming to improve HL may enhance their self-management practices and future outcomes. Quotes on the ‘Impact’ module can be found in Table 1-5.
- **3-2 f. Crosscutting Factors**: This module is comprised of diverse factors that are applicable to all four HL INPUT domains. These factors include, but are not limited to, cultural beliefs/values, personality, and self-efficacy. We also noticed the importance of a person’s cognitive capacities, socio-economic status, physical disability, social skills, motivation/need, prior knowledge, and disease management experience from previous encounters with the health care system. Similarly, community/cultural norms and beliefs may motivate or inhibit a person to engage actively in self-management practices. A researcher stated, “*There are numerous factors influencing a person’s decision to integrate the obtained information and services into their lifestyle, such as beliefs and readiness for change and motivation.*” Many patients were concerned about being stigmatized in the health care system, because they had previous difficulty expressing themselves due to language barriers, accent or inability to understand and felt embarrassed to ask questions. A patient indicated, “*… you don’t even want to voice your symptoms because first of all it’s not going to lead you anywhere and secondly, people [care providers] humiliate you.”* Patients mentioned several specific motivators (cues to action) that help provoke them to seek needed information or apply the obtained information in their disease management or behavioural change process. These include exacerbations or worsening of symptoms, fear (of what could happen), self-motivation, and external motivation (support from community or system). A patient expressed that *“I need to have the external bond [network] to use the exercise plan [pulmonary rehabilitation program] if they [other patients] do it as well …*” Another patient confirmed this point by stating: *“… a peer group … would help too.”* Quotes on the ‘Crosscutting Factors’ module can be found in Table 1-6.

## Discussion

There has recently been an increased emphasis placed on addressing the relationship of HL skills as they relate to the management and outcomes, among CAD patients. Evidenced by the literature, various individual and social factors influence a person’s willingness and capability to act on and involve in self-management of their chronic condition [44, 45, 71, 73]. To comprehensively qualify the multitude of influences on a patient’s HL, it was deemed critical to consider social support, culture, language, cognitive/physical factors, and demographic characteristics; including ethnicity, education, gender, and age as potential determinants of health outcomes [74–76].

Our proposed CADMaHL model aims to address the gaps identified in the literature by providing a conceptual framework of HL that allows health care professionals to empower their patients to follow optimal disease self-management practices to improve outcomes. To conceptualize HL in CAD management, our model considers the role of individual attributes and health system factors in the empowerment process through enhanced consideration for internal and external factors influencing CAD health outcomes. The model likely has the capacity to be both practical and applicable in real-world health contexts due to the involvement of patients, health care professionals, and policymakers in the development process. The model will work to maximize the successful interaction of personal capacity/skills, proper communication between patient and care provider, and social supports to improve HL; these interactions may in turn enhance disease self-management practices. The CADMaHL model also considers the effects of internal factors, such as person’s beliefs, attitude, worldview, cognition, and psychological issues on the decision-making process and how these factors may influence a patient’s effective interaction with the health care system [11, 77]. In addition, the assessment of coexisting cognitive deficits, common to patients with COPD, is neglected in current HL models, and should be included in a comprehensive model [73, 78–80]. Thereby, using an immediate caregiver at home, as a collateral source to help with this issue, could be beneficial [81, 82].

Although the CADMaHL model focuses on the role of HL in chronic airway disease management, our model’s development process and the integrated factors may be applicable to HL conceptual models for other chronic diseases as the main concepts and domains of HL are consistent across diseases and disease management. Additionally, the care process and necessary management capabilities are similar across different chronic diseases. Through our comprehensive approach, we gained valuable knowledge through many sources, including a systematic review of CAD HL measurement tools, patient-centred focus groups regarding HL barriers, key-informant interview on self-management strategies, and national respirologists perspectives on many topics. Involving multiple approaches (e.g., reviews, focus groups, interviews) and stakeholder groups (e.g., patients, researchers, clinicians) may provide researchers with a more robust understanding of myriad factors influencing patients’ disease management. Next, researchers of chronic diseases may consider the role of non-disease-specific factors included in our model, both internal (e.g., beliefs, attitudes) and external (e.g., socio-environment, health system factors). Researchers should also consider disease-specific barriers to optimal management, for example, cognitive deficits of individuals with COPD.

Other studies have concluded that empowering patients by improving their HL could enhance their self-efficacy in controlling disease symptoms and managing their disease condition [83–86]. To feel empowered in the disease management process, patients must be confident in their ability to apply the necessary skills, while navigating the complexities of the health care system [84, 85]. As seen in the literature, an empowered individual is more likely to comprehend their health issues and understand health instructions, seek proper care services, navigate successfully through the health care system, evaluate the usefulness of health information received, describe symptoms and triggers, and make informed decisions by applying the gained information and experiences to maintain their health [58, 59, 86]. With the goal of improving HL through empowering patients to properly manage their health condition, all the necessary factors that play crucial roles in this process should be identified and applied in the development of a HL model that may inform interventions aiming to improve disease self-management.

Previous HL models have described an individual’s capability to find and act on obtained information as the main contributors to health outcomes [87, 88]. However, investigators are beginning to report that HL is a shared responsibility between patients and care providers, and suggest including the complexity of the health care system in conceptualizing a HL model [89–91]. A HL researcher among our key-informants echoed this sentiment: “*Most of the HL work myopically focuses on the patient side. It’s time to focus on health care professionals and the system’s health literacy...measure both sides of the partnership...*” The findings from this study provide additional evidence that HL is influenced by the quality of patient and care provider interactions, and the availability and accessibility of information resources and treatment procedures that are appealing and applicable to the patient. We also identified one’s willingness and motivation (according to the health promotion concepts) to engage in self-management of their chronic disease as crucial factors to be considered. Finally, we conceptualized HL in a way to explain a structurally health literate competent care system, “*a system that adopts HL as an organizational value strategy in its care model”* [39, 89]. Such a care system should not only provide equal opportunity to all community members in accessing the needed health information and services, but also empower patients with the skills necessary to navigate the system and obtain the care they need to manage their condition [41, 66]. Health care providers should consider the role that they play in their patients’ HL, and how their behaviours across the core HL domains (e.g. communication, access, understand) affect each [92].

The CADMaHL model expands the current concept of HL to incorporate a multitude of factors that have been previously unconsidered within other existing models. The model incorporates critical factors such as social support, navigation and numeracy skills, and community and cultural norms that support, enable, or restrict the performance of disease self-management practices.

### Dual application of the proposed HL model

The CADMaHL model has been designed to facilitate more effective HL assessment and intervention studies for CAD patients.

- 4-1a. AssessmentOur CADMaHL may inform the development of HL measurement tools that can assess disease self-management practices, which can help clinicians and researchers to determine how their patient/client performs in disease self-management, what the difficulties in performing tasks and actions are, and what practical approaches are needed to improve potential gaps. Our model emphasizes collecting information about not only HL, but also the psychological, cognitive, behavioural, and systemic factors that may affect patient/client performance. Such information can help clinicians and health promotion researchers identify the skills, tasks, and factors that may serve as enablers or barriers to performing disease management practices.
- 4-1b. Intervention: We have developed a multi-dimensional framework that addresses a variety of HL factors across the core five domains and numeracy, which may allow for more effective and holistic HL interventions. In our model, HL interventions can be viewed as a process of patient-centered strategies that engage patients, caregivers, and care providers to develop educational resources that would enable successful disease self-management practices and navigation of the health care system. The concepts contained within our model will allow for patient-oriented HL interventions that empower patients to improve their health status by considering the internal factors, cultural/community norms and values, social support, and complexity of accessing and using health information and care services during the design and implementation of such interventions. In addition, the interventions should emphasize the tasks or actions that are required for patients to take, aiming for optimal disease self-management performance. Finally, our tool may serve as a knowledge repository for professionals across many different HL-related fields. As advocacy for HL is broad in its scope, engaging all levels of decision makers from sector-specific policy makers, to educators, to leaders of professional organizations and to the public at large, should be an important element of creating a health literate competent care system [86, 88, 92]. An important instrument in these efforts is the media [93, 94]. Efforts must be made to engage both conventional and social media since the ubiquity of chronic disease and the rapidly increasing coverage of its determinants, consequences. and management provide a ready-made venue for integrating the narrative about the role of health literacy in care services.

### Strengths and limitations

The strength of this model is its consideration for the complexity of the disease self-management process including previous knowledge, personal capabilities and attributes, the health care system and its complexities (systemic), all while building upon the five core HL domains and numeracy. In addition, we involved patients, HL researchers, and health care professionals in the conceptualization and development process of our multifaceted CADMaHL model from the earliest stages of the project. These key-stakeholders represented a global knowledge base and provided a wide variety of perspectives and leading-edge expertise. Their engagement allowed us to develop a model that may serve to inform holistic HL interventions and tools. Future research and application of the proposed model should examine its applicability to determine if this model is effective in designing HL interventions to empower patients, improve health outcomes, and reduce health care expenditures, as outlined in the framework (Fig. 1).

A limitation of our study is that our proposed model has not been tested yet; therefore, less is known on how it might guide the practice of clinicians as related to HL issues and airway disease self-management practices among patients. However, our model was developed with the engagement of patients and global key-informants in multiple stages of the study, and thus, it strived to encompass all angles and critical players in disease management, and is likely representative and applicable to current research and clinical practice in Canada.

## Conclusion

The knowledge gained from our previous research and the literature helped us to identify existing disease management barriers and gaps to HL in CAD patients. Such knowledge and application of a validity driven approach facilitated the development of a model that integrated various internal and external factors affecting a patients’ HL into one comprehensive framework, which other current HL conceptual frameworks fail to do. Our novel model incorporates feedback and perspectives from various key-stakeholders including health care professionals, policy makers, educators, and patients. Inclusion of internal factors, complexity of the health care system, and the multiple tasks, functions, and abilities that are necessary for a patient to actively perform disease self-management are the main strengths of the proposed model. We believe our model can help researchers develop more applicable screening and measurement instruments for assessing a CAD patient’s HL level, knowledge and abilities, and skills necessary to manage their chronic disease. Understanding and addressing a patient’s HL may enhance the quality of care and disease management. Application of the new knowledge could result in improved patient–care provider communication, improved understanding of an individual’s needs by clinicians, and improved educational resources and services that are accessible and understandable by the patient. Next, the anticipation is that the model may also guide the development of intervention studies to address the longitudinal influence of HL skills on CAD outcomes, a current gap in the literature. In addition, our approach to model development may be applicable to other chronic conditions. Collectively, outcomes and impacts can improve chronic airway disease management and health outcomes.

## Supplementary Information

**Additional file 1.** Questions from Focus Groups with Adult Asthma and COPD Patients.

## Data Availability

The data collected and analyzed for the current study are available from the corresponding author on reasonable request.

## Abbreviations

HL: Health literacy
CAD: Chronic airway disease
CADMaHL: Chronic Airway Disease Management and Health Literacy
COPD: Chronic obstructive pulmonary disease
NVivo: Navigating viewpoints, images and value observed
