# Supplementary material for: A conceptual model of functional health literacy to improve chronic airway disease outcomes
Source: BMC Public Health. 2021 Jan 30;21:252. doi: 10.1186/s12889-021-10313-x (PMC7847605; doi:10.1186/s12889-021-10313-x)
Supplement: Supplementary file 1 — Additional file 1. Questions from Focus Groups with Adult Asthma and COPD Patients. [file 12889_2021_10313_MOESM1_ESM.pdf]

Questions from Focus Groups with Adult Asthma and COPD Patients

QUESTION 1
How much information about health do you need to be able to make decisions about your disease?
QUESTION 2
Which source of information do you turn to when you are looking for or you want information about your asthma/COPD?
QUESTION 3
Share your experiences in searching/looking for information in relation to your asthma/COPD, in terms of accessibility and availability.
QUESTION 4
Do you have any examples of cases when you had difficulties understanding information about your asthma/COPD?
QUESTION 5
Besides your doctor, do you look for information elsewhere, and if so, what is your approach?
Follow-up: What about when you face something you don't understand about your asthma/COPD?
QUESTION 6
What would help you better understand the information you've received from different sources about your asthma/COPD?
QUESTION 7
What factors do you consider important to help you judge whether the information is useful for you?
Follow-up: What do you base your judgement on to determine that the information is useful?
QUESTION 8
What are the factors that help you trust the information you receive?
QUESTION 9
What would encourage you to use the information you receive from your doctor, other medical professionals, or other sources (e.g., Internet) to manage your disease?
QUESTION 10
What are your goals in relation to managing your disease, and what could help you achieve your disease management goals more easily?
QUESTION 11
What is/are the most common challenge(s) you face in managing your asthma/COPD?
QUESTION 12
Tell us about the skills you need to obtain and understand the information, in order to better manage your disease.
QUESTION 13
Where do you usually ask questions about your health (from your doctor, other medical professionals, or the Internet)?
QUESTION 14
Do you have trouble communicating with your health care professional about your health beliefs and values? Please explain.

Questions from Interviews with Health Literacy Key-informants (Health Care Professionals, Researchers, and Policymakers)

1. How would you define health literacy in chronic disease management?
2. In the proposed measurement tool for Health Literacy for asthma and COPD management, we have included five variables: (a) access, (b) understanding (comprehension) (c) evaluation (d) communication, and (e) using health information to make informed decisions. Which of these variables do you think need to be given a higher priority in developing a program or intervention to improve low health literacy skills?
3. Are there any more elements or domains that you think should be included in our measurement tool?
4. In your opinion, what skills does an individual need to successfully navigate the information provided in the health care system?
5. Can you provide some examples of barriers to chronic disease management that are imposed on patients due to limited knowledge and low health literacy skills?
6. As a care provider/professional or scientist, what are some of the unique challenges that your patients/clients may have come across in accessing health information?
7. Can you identify some barriers that inhibit clear communication of health information to patients/clients with limited literacy skills?
8. Were there instances where your patients/client had difficulty in understanding the information you provided?
9. How do you know when your patient/client does not understand the health information given, or when did you know you had to explain or elaborate on concepts?
10. What would help to make it easier for your patients/clients to understand the information given to them in regards to their health and health literacy?
11. What are the challenges you may face in providing services to diverse patient populations? i.e., differences in regards to culture, ethnic background, gender, age, education, etc.
12. What are some actions that you normally take to address these challenges?
13. In your opinion, how can you improve your patient/client's self-empowerment in order to help them become health literate?
14. In your opinion, what factors would help in making your patients clients confident about the trustfulness and relevancy of the information that they receive?
15. In your opinion, why do some people not using information they have received about health, despite them potentially knowing that the information they received might be helpful?
16. In your opinion, what would promote your patients/clients to use the information they have received about their health in their routine life?
17. In your opinion, who should be responsible for carrying out the health literacy programs and activities/interventions in healthcare system?
18. In your opinion, what regional, provincial and national strategies/approaches can improve population health literacy?

Questions from Interviews with Canadian Respirologists

1) What are the optimal disease management skills that an individual with asthma and/or COPD should have to properly manage their condition?

2) What are the most relevant disease management topics that should be included within a health literacy measurement tool for individuals with asthma and COPD, respectively?
